# Ovine Pulmonary Adenocarcinoma: A Unique Model to Improve Lung Cancer Research

**DOI:** 10.3389/fonc.2019.00335

**Published:** 2019-04-26

**Authors:** Mark E. Gray, James Meehan, Paul Sullivan, Jamie R. K. Marland, Stephen N. Greenhalgh, Rachael Gregson, Richard Eddie Clutton, Carol Ward, Chris Cousens, David J. Griffiths, Alan Murray, David Argyle

**Affiliations:** ^1^The Royal (Dick) School of Veterinary Studies and Roslin Institute, University of Edinburgh, Edinburgh, United Kingdom; ^2^Cancer Research UK Edinburgh Centre and Division of Pathology Laboratories, Institute of Genetics and Molecular Medicine, University of Edinburgh, Edinburgh, United Kingdom; ^3^School of Engineering and Physical Sciences, Institute of Sensors, Signals and Systems, Heriot-Watt University, Edinburgh, United Kingdom; ^4^School of Engineering, Institute for Integrated Micro and Nano Systems, The King's Buildings, Edinburgh, United Kingdom; ^5^Moredun Research Institute, Pentlands Science Park, Midlothian, United Kingdom

**Keywords:** human lung cancer, jaagsiekte sheep retrovirus, ovine pulmonary adenocarcinoma, sheep lung cancer models, comparative oncology

## Abstract

Lung cancer represents a major worldwide health concern; although advances in patient management have improved outcomes for some patients, overall 5-year survival rates are only around 15%. *In vitro* studies and mouse models are commonly used to study lung cancer and their use has increased the molecular understanding of the disease. Unfortunately, mouse models are poor predictors of clinical outcome and seldom mimic advanced stages of the human disease. Animal models that more accurately reflect human disease are required for progress to be made in improving treatment outcomes and prognosis. Similarities in pulmonary anatomy and physiology potentially make sheep better models for studying human lung function and disease. Ovine pulmonary adenocarcinoma (OPA) is a naturally occurring lung cancer that is caused by the jaagsiekte sheep retrovirus. The disease is endemic in many countries throughout the world and has several features in common with human lung adenocarcinomas, including histological classification and activation of common cellular signaling pathways. Here we discuss the *in vivo* and *in vitro* OPA models that are currently available and describe the advantages of using pre-clinical naturally occurring OPA cases as a translational animal model for human lung adenocarcinoma. The challenges and options for obtaining these OPA cases for research purposes, along with their use in developing novel techniques for the evaluation of chemotherapeutic agents or for monitoring the tumor microenvironment in response to treatment, are also discussed.

## Human Lung Cancer

Lung cancer is the most commonly diagnosed cancer in the world, with ~1.8 million new cases and 1.6 million cancer-related deaths recorded each year (1). Lung cancer treatment can be challenging as most patients are diagnosed when the disease is at an advanced stage. Poor response rates to radio-and chemotherapy have meant that overall 5-year survival rates are only 15%. The disease is highly heterogenous and is divided into several subtypes; their classification is under periodic review and in 2011 a multidisciplinary classification system was proposed by the European Respiratory Society and International Association for the Study of Lung Cancer (2). Their classification was based on factors such as disease biology, pathogenesis, and histopathology, which rendered terms such as bronchioloalveolar carcinoma (BAC) and it's mucinous and non-mucinous forms redundant.

Lung cancer is broadly classified into small-cell lung cancer, originating from bronchial neuroendocrine cells, and non-small cell lung cancer (NSCLC), originating from lung epithelial cells. NSCLC accounts for ~80% of cases and is subdivided into adenocarcinomas, large-cell carcinomas, squamous cell carcinomas, mixed, and undifferentiated tumors (3).

Adenocarcinomas are the most common form of lung cancer, accounting for 40% of cases. Hyperplasia of lung epithelial cells is thought to be the earliest cellular change that occurs in adenocarcinoma tumourigenesis. Termed “atypical adenomatous hyperplasia,” these pre-malignant lesions can accumulate cellular genetic abnormalities causing the cells to become pleomorphic, demonstrating a non-invasive, lepidic growth pattern along alveolar walls (4). Although these growths are known as adenocarcinoma-*in-situ*, complete surgical resection of lesions <30 mm in diameter results in almost 100% of cases gaining 5-year disease-free survival. However, if untreated, these lesions develop into invasive adenocarcinomas. Minimally invasive adenocarcinomas are lesions <30 mm in diameter with an invasive component <5 mm; surgical resection of these lesions is still likely to give an excellent prognosis. The cellular growth pattern (lepidic, acinar, papillary, or solid) is used to classify invasive adenocarcinomas >30 mm in diameter; these invasive forms are the most common clinical and pathological presentation of the disease. Lepidic-predominant adenocarcinoma describes invasive adenocarcinomas that have a predominant lepidic pattern with an invasive component >5 mm (previously termed non-mucinous BAC). In addition, a mucinous form of lepidic adenocarcinoma may also be encountered (previously termed mucinous BAC); this non-invasive, minimally-invasive or invasive disease is often bilateral and multifocal with extensive mucous production. Patients suffering from this subtype present with a cough and extensive mucous production that can lead to death from respiratory failure without any evidence of invasive disease (2).

## Mouse Models of Human Lung Cancer

Numerous animal models (primates, dogs, hamsters, mice) have been described for lung cancer research (5, 6). Mice have traditionally been considered the preferred model due to cost-effectiveness and ease of genetic manipulation (7). Many mouse models are now available, including inbred strains exhibiting high rates of spontaneous lung tumors (8–10) (useful for chemoprevention studies), chemical (11)/carcinogen (5)/environmental-induced lung cancer models (12) (allowing the study of tumor initiation and progression) and orthotopic xenograft models (13–16) (facilitating the analysis of both primary and metastatic tumors). Hundreds of transgenic mouse strains which incorporate the genetic mutations that occur in human lung cancer can now be produced. These mice will produce tumors with greater similarity to human disease and allow the genes that drive lung cancer development and progression to be identified (17). These genetic changes include tumor suppressor gene inactivation (p53, retinoblastoma, and p16), oncogene activation (K-ras), altered growth factor expression (18), loss of heterozygosity, and amplification of specific chromosomal regions (17, 19). The use of bioluminescent or fluorescent reporters in mice is also possible (20, 21). These models allow lineage tracing to be performed and can lead to the identification of individual oncogenes involved in tumourigenesis and can enable the determination of the tumor cell type origin (22).

Despite these advantages, murine models do not accurately represent the advanced stages of lung cancer and are poor predictors of clinical outcome. Each model also has its own specific disadvantages, such as a lack of metastasis in genetic and chemically induced models and the inability to examine immune response in tumor development/progression in xenograft models that require the use of immunodeficient mice (7). The perceived advantages of having multiple models can also be seen as a limitation, as no one single model can be used to examine all stages of the disease.

## Comparative Human and Sheep Pulmonary Anatomy and Physiology

**Figure 1:**
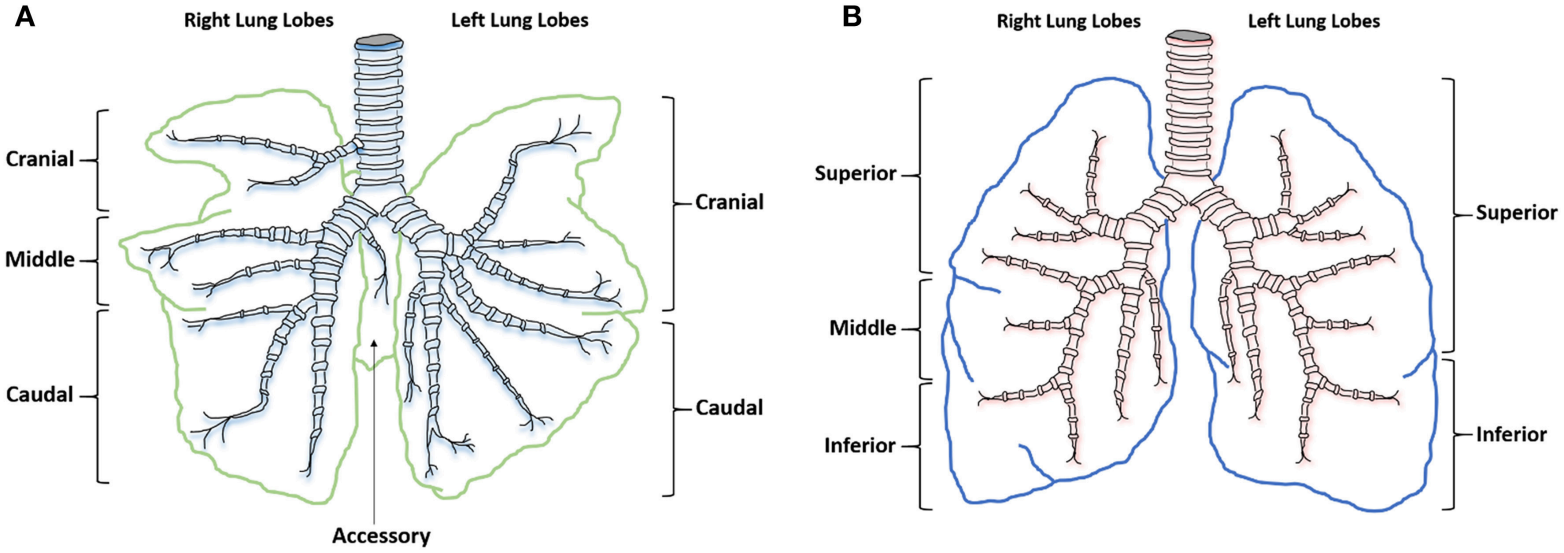
Ovine and human comparative gross anatomy. **(A)** Ovine lower respiratory tract. **(B)** Human lower respiratory tract.

Similarities between human and sheep pulmonary anatomy and physiology has led to sheep being identified as an excellent model for investigating human lung function and disease. Human lung anatomy consists of the left lung divided into superior and inferior lobes and the right into superior, middle and inferior lobes. Sheep anatomy is similar with the left lung divided into cranial and caudal lobes and the right into cranial, middle, caudal, and accessory lobes. In sheep each lobe is separated by tissue septa, which limits lobular connectivity (23) (Figure 1). Although in sheep the right cranial lobe bronchus arises directly from the trachea before the tracheal bifurcation (24), with respiratory bronchioles that are poorly developed (23) the remaining tracheobronchial tree is similar in both species, showing an irregular dichotomous branching pattern. The distribution of differentiated respiratory epithelial cells (25), mast cells (26), and airway smooth muscle (27) is also comparable between the species. Although human lungs have fewer intravascular macrophages compared with the large number seen in sheep lungs (28), increased numbers can occur after an endotoxic insult. Lung development is also similar between the species; lamb lungs show significant similarities to human infant lungs, including prenatal alveologenesis, airway branching patterns, bronchiolar club cell number, type II alveolar epithelial (pneumocytes) development, and the presence of airway submucosal glands (29).

Similarities in lung size allow sheep models to be used in ways not available in mouse models; techniques including drug administration, advanced imaging (30), ultrasound (31), endoscopy, and surgical procedures can be used in sheep as they would in humans (32). With the correct animal handling facilities, where appropriate, procedures can be performed in conscious or minimally sedated animals, rather than using general anesthesia. Repeated blood sampling and tissue collection is easier in sheep and their longevity allows chronic conditions to be modeled, while also enabling the evaluation of long-term treatments. These factors make sheep excellent models for human respiratory conditions (24) such as asthma (33), cystic fibrosis, chronic obstructive respiratory disease (34), respiratory syncytial virus infection (35), and now cancer (36).

## Ovine Pulmonary Adenocarcinoma

Ovine pulmonary adenocarcinoma (OPA) is a neoplastic lung disease caused by the jaagsiekte sheep retrovirus (JSRV) (37–40). This betaretrovirus is the only known virus capable of inducing the formation of naturally occurring lung adenocarcinomas. Since the disease was first described in South Africa in the nineteenth century (41), JSRV infection has been identified in numerous sheep breeds and small ruminants throughout the world, the virus however has never been shown able to infect humans (42, 43). Although natural JSRV infection can occur in goats this rarely results in tumor formation and experimental infection of goat kids induces tumors with a different macroscopic and histological appearance to those seen in lambs (44). OPA is endemic in the UK and represents a major economic and animal welfare concern (39, 45). Within-flock disease incidence levels can be as high as 30%, although levels of 2–5% are more common (46). Mortality rates of 50% can be seen following initial disease identification within a flock (47); however, as the disease becomes endemic rates reduce to 1–5% (41, 48). Disease transmission occurs predominantly through the aerosol route (41, 47, 49), meaning close contact with infected sheep is a significant risk factor. The virus has been detected in the milk and colostrum of infected ewes, which poses a potential source of infection for new born lambs (50).

## JSRV Biology

JSRV particles contain two copies of single-stranded positive sense RNA. It's genome of ~7,460 nucleotides contains four genes encoding viral proteins (39). These four genes are: *gag* (encoding the matrix, capsid, and nucleocapsid proteins); *pro* (encoding aspartic protease); *pol* (encoding reverse transcriptase and integrase enzymes); and *env* (encoding surface and transmembrane envelope glycoproteins) (51, 52). An additional open reading frame, known as *orfX*, which overlaps with the *pol* gene, has also been identified; however, it is not required for *in vitro* cellular transformation (53) or *in vivo* oncogenesis (54–56). Interestingly, JSRV-induced neoplastic transformation is mediated by the viral Env glycoprotein, although the mechanisms underlying this process are not completely understood. The transforming activity of Env was first shown *in vitro* using rodent fibroblasts (53, 57), with subsequent *in vivo* experiments showing that the administration of viral vectors expressing Env to the lungs of mice (56) and sheep (55) results in adenocarcinoma formation. Env localization at the plasma membrane may enable it to interact with other molecules such as protein kinases (58), leading to the activation of downstream pathways that promote cellular proliferation and survival. The Ras-MEK-ERK (59, 60) and PI3K-AKT-mTOR (59, 61, 62) pathways are commonly activated in OPA tumors; others may include EGFR, RON-HYAL2 and heat shock proteins (63). Following pathway activation, it is likely that further mutations are required for tumors to develop, such as telomerase activation (62), the activation of other cellular oncogenes or the inactivation of tumor-suppressor genes. For a detailed description of JSRV structure and replication cycle see the recent review by Youssef et al. (36).

## Endogenous Retrovirus and Immune Responses

Endogenous retroviruses are viruses that have become integrated into host germ-line DNA and are passed through the generations. The sheep genome contains numerous endogenous JSRV (enJSRV) related proviruses with over 90% sequence similarity to exogenous JSRV (exJSRV) (64, 65). These enJSRV proviruses are not oncogenic (they lack the oncogenic Env c-terminal domain present in exJSRV) (37, 51, 66, 67), but are transcriptionally active, with studies showing viral RNA and protein expression in the female reproductive tract and in fetal tissues (67, 68). The expression of these viral proteins may help protect the host from exJSRV infection, either by receptor competition or through the prevention of exJSRV viral particle transport and cellular exit (68, 69).

JSRV infection lacks a specific cellular or humoral immune response to viral proteins. Although neutralizing antibodies specific for JSRV have been found in a minority of infected animals (44, 70), the lack of a consistent adaptive response is likely due to sheep being immunologically tolerant of JSRV antigens as a result of the expression of enJSRV proteins in the fetal thymus during T lymphocyte development. Tumor cells also downregulate the expression of class I antigens of the major histocompatibility complex, preventing their recognition by CD8^+^ T lymphocytes. The influx of alveolar macrophages following JSRV infection, which produce large amounts of interferon gamma, also fails to activate T cells or produce a JSRV-specific immune response. Overproduction of surfactant proteins in OPA is also proposed to contribute to the absence of an effective immune response (71).

## OPA Histology and Comparison With Human Lung Adenocarcinomas

**Figure 2:**
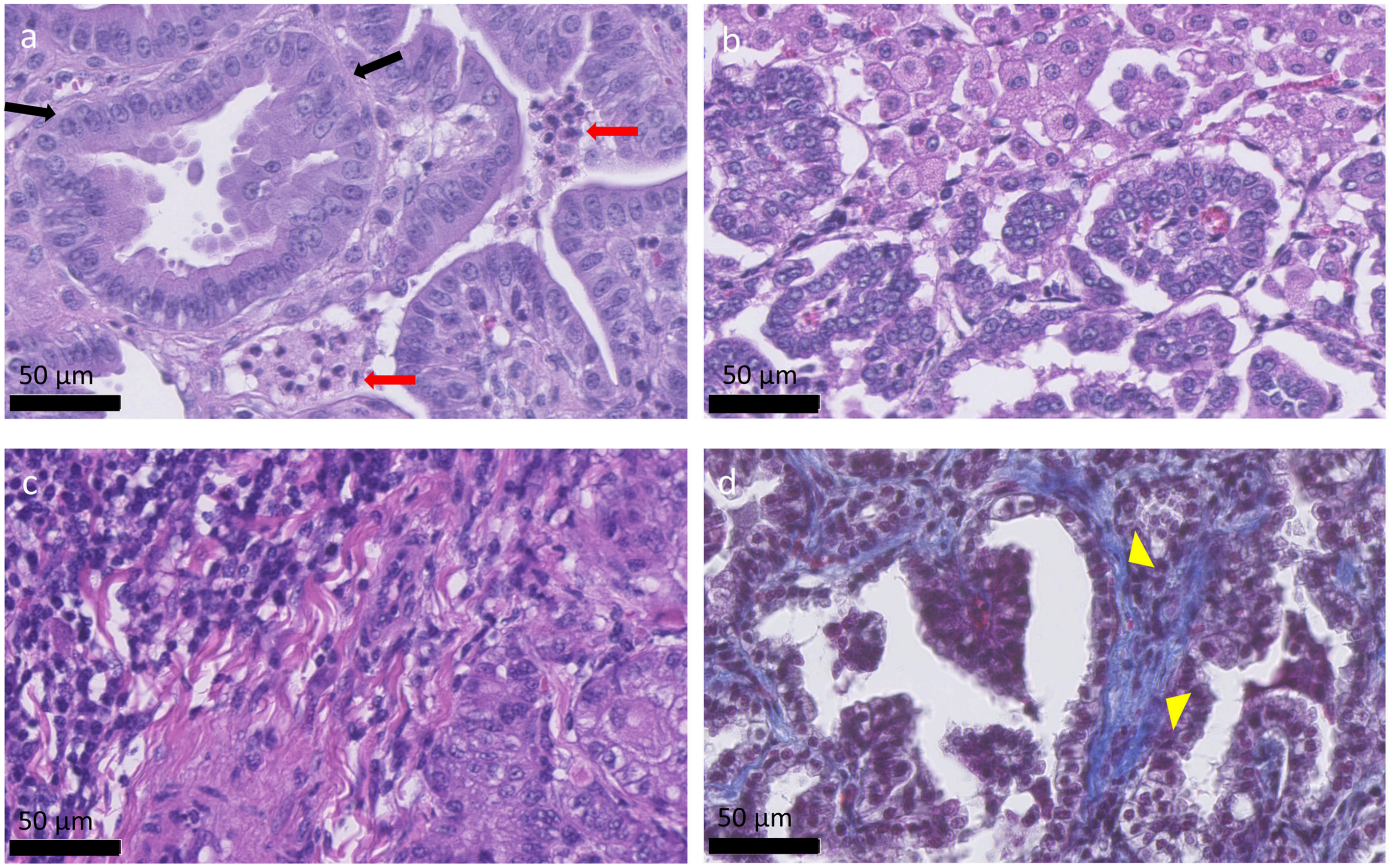
Histological appearance of OPA tumors. **(a–c)** OPA haematoxylin and eosin stained sections. **(a)** Columnar tumor cells can be seen lining the alveolar septa (black arrows), forming acinar, or papillary proliferations. Two groups of neutrophils are present between the tumor cells (red arrows). **(b)** Alveolar macrophages can be seen at the top right-hand side of the image and are characterized by large amounts of foamy cytoplasm. **(c)** Accumulation of mononuclear immune cells, mostly lymphocytes, and plasma cells, can be observed at the top left-hand side of the image. **(d)** Masson's trichrome stained section. Collagen is stained blue and can be identified surrounding the tumor cells (yellow arrow heads), acting as a scaffold for the influx of inflammatory cells.

OPA tumors are composed of non-encapsulated neoplastic foci originating from JSRV infected and transformed bronchiolar and alveolar secretory epithelial cells (72, 73). Type II pneumocytes are the predominant cell type, with smaller numbers of bronchiolar club cells and undifferentiated cells present (74). Type II pneumocytes function to synthesize, store, and secrete alveolar surfactant, whereas bronchiolar club cells produce protein components that line the extracellular surface of bronchioles. Tumor cells are typically cuboidal or columnar, with or without cytoplasmic vacuolation while also exhibiting a low mitotic rate. However, other tumor areas may show higher degrees of malignancy with high mitotic rates and areas of necrosis (74, 75). Fibrovascular connective tissue surrounds tumor cells and acts as a scaffold for the influx of inflammatory cells. Large numbers of macrophages are typically identified (71); however, neutrophil number can vary depending on the presence of a bacterial co-infection (Figure 2). Tumor cell proliferation initially occurs along alveolar septa (lepidic growth), before extending into bronchioles through the formation of acinar or papillary proliferations. Infected cells release JSRV virions which spread within the lung forming new foci of infection, resulting in a highly oligoclonal tumor (76). Neighboring tumor foci eventually expand and coalesce to form a single large tumor. Intrathoracic and extrathoracic metastasis is possible and has been identified in ~10% of cases (77–80).

Although early reports detailing OPA described the disease as having similarity to human BAC, under the current human lung classification system early OPA lesions would fit a description of a minimally invasive adenocarcinoma or lepidic-predominant adenocarcinoma; whereas typical advanced lesions would more closely resemble adenocarcinoma with a papillary or acinar-predominant growth pattern. Importantly, OPA has the greatest similarity to the rare multifocal, non-invasive presentation of human lung adenocarcinoma (such as the mucinous forms), and is less similar to the more common aggressive, metastatic forms of the disease (36).

## Experimental Systems for Studying OPA

An *in vivo* sheep model was the first reproducible experimental system developed to study OPA. Initial studies showed that the injection of OPA tumor homogenates or JSRV purified from lung fluid, into the trachea of healthy sheep, led to the appearance of lung tumors (81, 82). It was later shown that using neonatal lambs improved the rate of infection and decreased the time for tumors to develop (73, 83). Further refinement of the model has been achieved through cloning and sequencing of the JSRV genome (51, 84) and the generation of an oncogenic and infectious molecular clone, which has enabled virus production using *in vitro* transfection of cell lines (85, 86). A JSRV replication-defective virus (JS-RD) that expresses only the Env glycoprotein has also been used in the *in vivo* lamb model system (55). As this vector is replication defective, it can infect and transform target cells but cannot replicate further. As these transformed cells proliferate, they form well-isolated uniform neoplastic foci, each being a separate transformed focus. Therefore, tumors induced by JS-RD have a reduced degree of polyclonality compared to naturally occurring OPA and human adenocarcinomas. This reduced heterogeneity might add value to the experimental OPA model, as the effects of targeting specific pathways would be easier to identify.

The *in vivo* lamb model also has the potential for studying pathogenic mechanisms in early stage disease. This is important as human clinical tissue from early cases is generally unavailable. However, while the lamb model is useful for studying OPA from initial infection up to the formation of small tumors, for welfare reasons it is not appropriate to let the disease reach an advanced clinical stage. As such, naturally occurring cases are more suitable for studying more advanced disease stages.

Mouse OPA models are alternative *in vivo* systems that do not necessitate the use of large animal facilities. Using both immunodeficient mice (56) and immunocompetent mice models (87) studies have shown that the intranasal administration of adeno-associated virus vectors encoding JSRV Env leads to the formation of lung adenocarcinomas that are comparable to those found in sheep and humans.

The lack of a cell line that can support JSRV replication *in vitro* has limited the amount of *in vitro* research that has been performed on OPA (88). Some studies have therefore focused on the use of primary OPA tumor cells (62, 89, 90); however, extended *in vitro* culture of these cells typically leads to a cessation in virus production (89, 90). These alterations in JSRV expression can be either delayed or reversed when cells are cultured in a 3D environment (89, 91), indicating that 3D culture models may more accurately recreate the oncogenic events that occur in OPA. Lung tissue explants are another *in vitro* model that has been developed. These precision-cut lung slices are tissue discs 300 μm thick and 8 mm in diameter cut using an automated microtome (59, 92), and are thought of as a transitional model between the other *in vitro* and *in vivo* available systems.

## OPA as a Model for Studying Pulmonary Adenocarcinoma Tumourigenesis

It is not clear whether human pulmonary adenocarcinoma arises from a stem cell population that is able to differentiate into alveolar type II pneumocytes and bronchiolar club cells, from a lineage-specific progenitor cell, or from a fully differentiated cell type (93). In mice putative bronchioalveolar stem cells (BASC) have been identified which are proposed to be the cell type of origin of lung adenocarcinomas in response to oncogenic K-ras (94). However, the presence of BASC in humans and sheep has not been firmly established (95). Cells displaying some features of BASC have been described in sheep (72, 96) but their significance in OPA tumourigenesis remains unclear.

As described in the previous section, in the *in vivo* experimental lamb model, JSRV is able to induce the formation of OPA tumors with a short incubation period (82, 83). In contrast, adult sheep have been shown to be resistant to experimental induction of OPA (83). This age-related susceptibility to OPA tumor formation is due, at least in part, to the availability of susceptible target cells capable of being infected and transformed. JSRV, like most retroviruses, infects dividing cells much more efficiently than non-dividing cells (73). Normal sheep and human adult lungs have relatively low rates of bronchioalveolar cell division. However, the lungs of both species are not fully mature at birth and continue to develop for a period of time resulting in an increase in alveolar number (97, 98). One study has shown that the cells targeted for JSRV transformation and tumourigenesis are proliferating progenitor cells of type II pneumocyte lineage, termed lung alveolar proliferating cells (LAPCs), rather than mature post-mitotic type II pneumocytes, bronchiolar club cells, or BASC. LAPCs are significantly more abundant in lambs compared to adult sheep, therefore the age-related susceptibility of OPA development is directly related to the abundance of LAPCs (73).

The adult lung has significant reparative capabilities despite the low proliferation rate of respiratory epithelial cells, LAPCs are proposed to play an important role in tissue repair following injury. Chemically-induced injury to the respiratory epithelium has been shown to increase the number of LAPCs in adult sheep, which subsequently rendered the sheep susceptible to JSRV infection and transformation (73). This may have relevance for naturally occurring OPA, as cases typically present with a variety of other parasitic, bacterial, or viral infections (45). Classically, these infections were considered as “secondary” to JSRV infection; however, it is possible that they are important factors that contribute to pulmonary inflammation and tissue damage that facilitate JSRV infection and tumorigenesis. In humans, recent studies have identified a subpopulation of type II pneumonocytes that exhibit properties of progenitor cells, including self-renewal and proliferation in response to injury (99, 100). Thus, OPA may have value as a comparative model for understanding the role of alveolar progenitor cells in carcinogenesis.

## OPA Diagnosis and Potential Sources of Experimental Animals

Although OPA has been identified in sheep <1 year old the majority of naturally occurring clinical cases are seen in sheep aged between 2 and 4 years of age. The diagnosis of clinical OPA can usually be based on clinical signs including pneumonia (non-responsive to antibiotic treatment), dyspnea, and tachypnoea (especially when herded) in combination with weight loss (despite maintaining a normal appetite) (101). Thoracic auscultation may be of benefit for diagnosing advanced cases, where adventitious lung sounds (crackles) can be heard over the majority of the lung fields due to the presence of fluid in the airways (102). Significant volumes of fluid draining from the nostrils is a pathognomonic clinical sign of OPA (103); at this stage tumors will typically occupy more than 30% of the lung volume (101). Although historically these advanced tumors were presumed to have developed over many months or years (101), new evidence shows that some OPA tumors may develop very rapidly (104).

Pre-clinical antemortem diagnosis is important not only for removing infected animals from flocks but also in identifying cases for experimental purposes; however, this diagnosis remains a significant challenge. Pre-clinical diagnosis based on a clinical examination is difficult as there may be a lack of adventitious lung sounds detectable by auscultation (105). Many infected sheep never develop clinical signs during their commercial lifespan (106), and those that do may only do so when the tumor is sufficiently large to compromise respiration. During this pre-clinical period these apparently healthy animals may be infectious and represent a source of infection for the rest of the flock.

**Figure 3:**
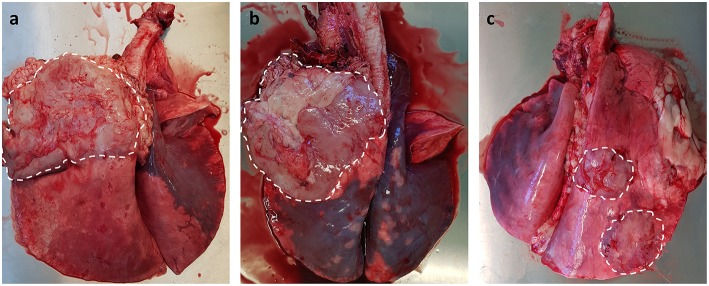
Gross pathology of OPA tumors. **(a,b)** Large single advanced OPA tumors affecting the entire left cranial lung lobe. The lesions are gray in color with a clear distinct boundary between neoplastic tissue and the neighboring pink aerated lung. Extensive fibrous tissue can be seen attached to the overlying pleura of the tumor. **(c)** Two discrete OPA tumors within the right cranial and caudal lung lobes.

As JSRV infected sheep fail to produce a significant humoral immune response to viral proteins (107), it has not been possible to develop serological diagnostic assays. Alternative diagnostic tests have been developed for virus detection in blood samples using PCR technology (108); unfortunately the numbers of virally infected blood mononuclear cells (monocytes, B and T lymphocytes) are very low, which results in high false negative results (109). Despite this significant limitation, the test can be used for identifying infected flocks rather than for testing individual animals. The same PCR technique has been employed to detect JSRV-infected cells in bronchoalveolar lavage samples (110), which offers better sensitivity than the blood test. However, this method requires sedation for sample collection, only tests a small region of the lung (potential for missing early cases) and does not lend itself to large-scale routine on-farm testing. Currently, the gold standard diagnostic test for both clinical and pre-clinical OPA remains gross pathology and histology performed at post mortem examination. OPA tumors can be extensive, involving the entire lung lobe, or may occur as multifocal discrete lesions. These lesions fail to collapse upon entering the thoracic cavity and can distort the normal architecture of the affected lung lobe, with clear boundaries between tumor tissue and adjacent pink aerated lung. Although the overlying pleura can remain intact, fibrinous adhesions between the visceral pleura and chest wall can be seen (Figure 3). Tracheobronchial and mediastinal lymph nodes usually appear grossly normal but may be enlarged in cases of metastasis or pneumonia (39).

**Figure 4:**
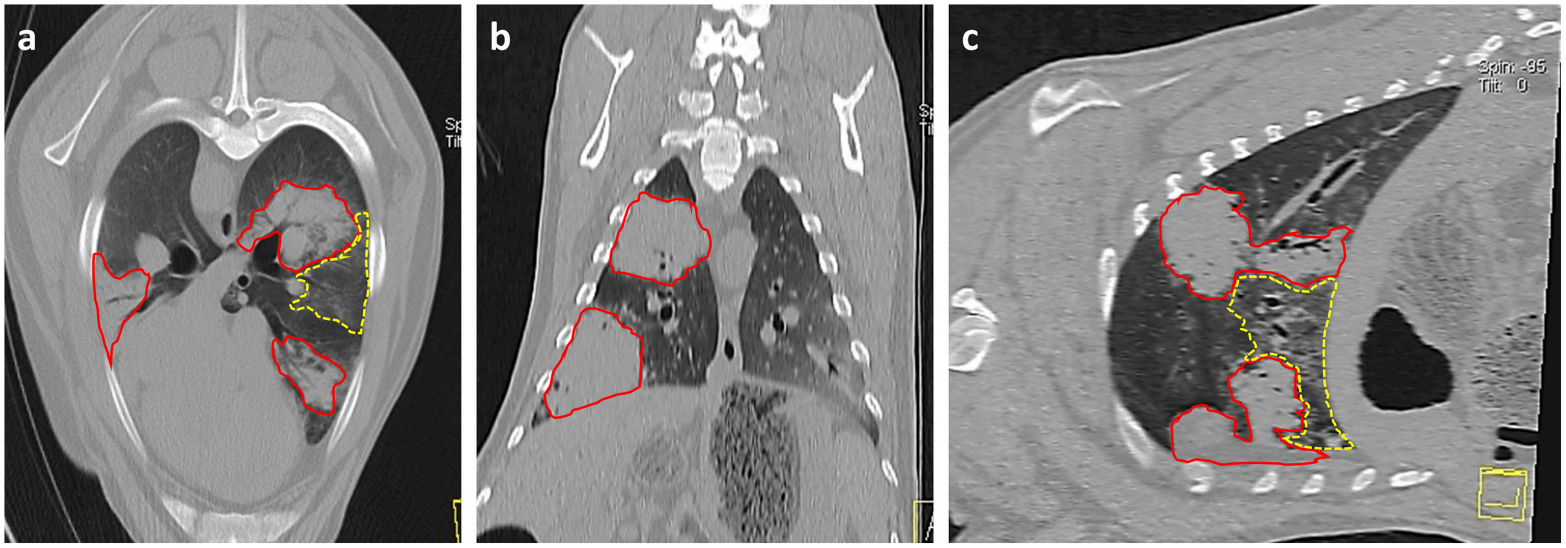
Thoracic CT images of OPA tumors**. (a)** Axial, **(b)** Coronal, and **(c)** Sagittal planes. Three large areas of increased radiopacity are seen within the lung lobes consistent with advanced OPA tumors (outlined in red). One lesion is present within the dorsal region of the left cranial lung lobe with a further lesion in the ventral region of the left caudal lung lobe. A smaller lesion is present within the right caudal lung lobe. A patchy and hazy area of increased opacity (ground glass appearance), with preservation of bronchial and vascular patterns, is present (outlined in yellow) between the two tumors in the left lung lobes. This area is consistent with regions of neoplastic foci or a secondary pneumonia.

Imaging modalities such as radiography and computed tomography (CT) have been suggested for use in OPA diagnosis. CT is considered the gold standard imaging modality for human lung parenchyma and has been used in studies to monitor the development and progression of OPA in both naturally occurring (111) and experimentally infected animals (70). CT will detect smaller lung lesions than can be identified using radiography, particularly if located in the ventral margins of the cranial lung lobes that are difficult to image using radiography (Figure 4). However, radiography and CT are cost prohibitive for commercial flocks and require specialized equipment and sedation/general anesthesia (101). Ultrasonography is an extremely useful imaging technique for OPA diagnosis and can be performed on-farm in conscious animals. With experience, the procedure can be performed in <1 min per sheep (112), can differentiate between chronic lung lesions and can detect OPA lesions as small as 1–2 cm in diameter involving the visceral pleura (31). One study conducted transthoracic ultrasound examinations of 100 sheep presented for the investigation of weight loss with or without respiratory signs; of these cases, 41 sheep were diagnosed as OPA positive based on ultrasound examination alone, with all cases having the diagnosis confirmed at post mortem. The remaining sheep had no ultrasonographic changes characteristic of OPA and had no gross OPA lesions at post mortem. The study demonstrated the high specificity of transthoracic ultrasound for OPA diagnosis in clinically affected animals, producing no false positive or negative results (31). Although a negative scan cannot guarantee that an animal does not have early OPA and/or is not infected with JSRV, it has been suggested that transthoracic ultrasound examination can be used to confirm a suspected diagnosis, screen flock replacements, and screen sheep in known OPA-affected flocks. It is also an ideal method for identifying pre-clinical cases for experimental use, as individual cases can be selected based on the size and location of OPA lesions.

## OPA as a Pre-Clinical Model

The use of OPA as a model for monitoring the tumor microenvironment, assessing the effectiveness of chemo- and radiotherapy or in the development of surgical techniques has not been previously documented. However, if techniques that are commonly used in the treatment of human lung cancer patients such as ultrasound, general anesthesia, CT, and surgery can be incorporated into the OPA model, this would further demonstrate its potential as an excellent translational research tool. One paper documented the use of naturally occurring OPA cases combined with CT evaluation, post mortem examination/histopathology, trace element, and liver enzyme activity analysis in a long-term study evaluating the impact of nutritional selenium on tumourigenesis and progression (111). This study demonstrated the potential for the OPA model to be integrated with multiple techniques to provide comprehensive information on tumor pathogenesis.

In terms of chemotherapy models, *in vitro* work using rat fibroblasts has shown that through AKT degradation, Hsp90 inhibitors can block the transformation and revert the phenotype of cells already transformed by JSRV Env. Hsp90 inhibitors can also reduce the proliferation of primary and immortalized OPA cell lines (63). The chemotherapeutic potential of agents such as Hsp90 inhibitors could be assessed using OPA cases if techniques could be integrated into the model to assess the tumors response to treatment.

One current ongoing multidisciplinary project that is using naturally occurring OPA cases as a pre-clinical translational model is the Implantable Microsystems for Personalized Anti-Cancer Therapy (IMPACT) programme at the University of Edinburgh (113). This project aims to develop novel miniaturized implantable oxygen and pH sensors that can monitor oxygen levels and pH within a solid tumor; the identification of hypoxic and acidic regions within a tumor can lead to more targeted therapies against these radiation and chemo-resistant regions. Functionality of these sensors is being validated following their implantation into OPA tumors using a CT-guided percutaneous method. This technique is similar to that used for transthoracic needle biopsies in human patients. If successful, then studies such as this will provide exciting new translational opportunities for the OPA model to be used in pre-clinical research (see accompanying article, Gray et al. manuscript submitted)^1^.

## Conclusion

As outlined here, OPA has great potential to be used as an excellent model for studying multiple aspects of human lung cancer biology. As a result, *in vivo* and *in vitro* OPA experimental models have been developed for the study of JSRV Env mediated oncogenesis; these have been successfully used to determine the molecular pathways involved in lung cancer pathogenesis. However, the potential for OPA to be used as a pre-clinical animal model for assessing human lung cancer treatment strategies has yet to be fully exploited. Naturally occurring OPA cases are readily available from infected flocks due to the endemic nature of the disease in many countries and pre-clinical cases can be identified by the use of ultrasound scanning programmes. The use of naturally occurring cases could decrease the use of experimentally induced OPA tumors in lambs, reducing ethical concerns with this model. Future studies that can integrate techniques commonly used in the treatment of human lung cancer patients, such as ultrasound, general anesthesia, CT, and surgery, would further strengthen the effectiveness of OPA as a pre-clinical cancer research model.

## Author Contributions

MG wrote the majority of the manuscript and composed the majority of the figures with contributions from JM who wrote the experimental systems for studying OPA. Critical revisions were made by MG, JM, PS, JRKM, SG, RG, RC, CW, CC, DG, AM, and DA. All authors read and approved the final manuscript.

### Conflict of Interest Statement

The authors declare that the research was conducted in the absence of any commercial or financial relationships that could be construed as a potential conflict of interest.

## Footnotes

^1^Gray M, Sullivan P, Marland JRK, Greenhalgh SN, Meehan J, Gregson R, et al. A novel translational ovine pulmonary adenocarcinoma model for human lung cancer.

## References

[1] Ferlay J Soerjomataram I Dikshit R Eser S Mathers C Rebelo M. Cancer incidence and mortality worldwide: Sources, methods and major patterns in GLOBOCAN 2012. Int J Cancer. (2015) 136:359–86. 10.1002/ijc.29210 25220842

[2] Travis WD Brambilla E Noguchi M Nicholson AG Geisinger KR Yatabe Y. International association for the study of lung cancer/american thoracic society/european respiratory society international multidisciplinary classification of lung adenocarcinoma. J Thorac Oncol. (2011) 6:244–85. 10.1097/JTO.0b013e318206a221 21252716 PMC4513953

[3] Sekido Y Fong KM Minna JD. Progress in understanding the molecular pathogenesis of human lung cancer. Biochim Biophys Acta. (1998) 1378:21–59. 9739759 10.1016/s0304-419x(98)00010-9

[4] Carey FA Wallace WA Fergusson RJ Kerr KM Lamb D. Alveolar atypical hyperplasia in association with primary pulmonary adenocarcinoma: a clinicopathological study of 10 cases. Thorax. (1992) 47:1041–3. 10.1136/thx.47.12.1041 1494768 PMC1021097

[5] Coggins CR A review of chronic inhalation studies with mainstream cigarette smoke, in hamsters, dogs, and non-human primates. Toxicol Pathol. (2001) 29:550–7. 10.1080/019262301317226357 11695572

[6] Liu J Johnston MR. Animal models for studying lung cancer and evaluating novel intervention strategies. Surg Oncol. (2002) 11:217–27. 10.1016/S0960-7404(02)00053-1 12450558

[7] Pozzi A Mouse Models of Lung Cancer, Principles and Practice of Lung Cancer. 4th ed. Philadelphia, PA: Lippincott Williams and Wilkins (2010), p. 179–187.

[8] Lynch CJ. Studies on the relation between tumor susceptibility and heredity: III. spontaneous tumors of the lung in mice. J Exp Med. (1926) 43:339–55. 10.1084/jem.43.3.339 19869127 PMC2131108

[9] Tuveson DA Jacks T. Modeling human lung cancer in mice: similarities and shortcomings. Oncogene. (1999) 18:5318–24. 10.1038/sj.onc.1203107 10498884

[10] Stoner GD Adam-Rodwell G Morse MA Lung tumors in strain a mice: application for studies in cancer chemoprevention. J Cell Biochem. (1993) 53:95–103. 10.1002/jcb.240531014 8412213

[11] Hoenerhoff MJ Hong HH Ton T-V Lahousse SA Sills RC. A review of the molecular mechanisms of chemically induced neoplasia in rat and mouse models in National Toxicology Program bioassays and their relevance to human cancer. Toxicol Pathol. (2009) 37:835–48. 10.1177/0192623309351726 19846892 PMC3524969

[12] Szymanska H Sitarz M Krysiak E Piskorowska J Czarnomska A Skurzak H. Genetics of susceptibility to radiation-induced lymphomas, leukemias and lung tumors studied in recombinant congenic strains. Int J Cancer. (1999) 83:674–8. 10.1002/(SICI)1097-0215(19991126)83:5<674::AID-IJC18>3.0.CO;2-M 10521806

[13] Mase K Iijima T Nakamura N Takeuchi T Onizuka M Mitsui T. Intrabronchial orthotopic propagation of human lung adenocarcinoma-characterizations of tumorigenicity, invasion and metastasis. Lung Cancer. (2002) 36:271–6. 10.1016/S0169-5002(02)00004-1 12009237

[14] Kozaki K-I Miyaishi O Tsukamoto T Tatematsu Y Hida T Takahashi T. Establishment and characterization of a human lung cancer cell line NCI-H460-LNM35 with consistent lymphogenous metastasis via both subcutaneous and orthotopic propagation. Cancer Res. (2000) 60:2535–2540. 10811136

[15] Kuo T-H Kubota T Watanabe M Furukawa T Kase S Tanino H. Orthotopic reconstitution of human small-cell lung carcinoma after intravenous transplantation in SCID mice. Anticancer Res. (1992) 12:1407–10. 1332577

[16] Manzotti C Audisio RA Pratesi G. Importance of orthotopic implantation for human tumors as model systems: relevance to metastasis and invasion. Clin Exp Meta. (1993) 11:5–14. 10.1007/BF00880061 8422706

[17] Kohno T Yokota J. How many tumor suppressor genes are involved in human lung carcinogenesis? Carcinogenesis. (1999) 20:1403–10. 10.1093/carcin/20.8.1403 10426784

[18] Moorehead RA Sanchez OH Baldwin RM Khokha R. Transgenic overexpression of IGF-II induces spontaneous lung tumors: a model for human lung adenocarcinoma. Oncogene. (2003) 22:853–7. 10.1038/sj.onc.1206188 12584565

[19] Salgia R Skarin AT. Molecular abnormalities in lung cancer. J Clin Oncol. (1998) 16:1207–17. 10.1200/JCO.1998.16.3.1207 9508209

[20] Hoffman RM. Green fluorescent protein imaging of tumour growth, metastasis, and angiogenesis in mouse models. Lancet Oncol. (2002) 3:546–56. 10.1016/S1470-2045(02)00848-3 12217792

[21] El Hilali N Rubio N Martinez-Villacampa M Blanco J Combined noninvasive imaging and luminometric quantification of luciferase-labeled human prostate tumors and metastases. Lab Invest. (2002) 82:1563–71. 10.1097/01.LAB.0000036877.36379.1F 12429816

[22] Mainardi S Mijimolle N Francoz S Vicente-Due-as C Sánchez-García I Barbacid M. Identification of cancer initiating cells in K-Ras driven lung adenocarcinoma. Proc Natl Acad Sci USA. (2014) 111:255–60. 10.1073/pnas.1320383110 24367082 PMC3890787

[23] Kirschvink N Reinhold P. Use of alternative animals as asthma models. Current Drug Targets. (2008) 9:470–84. 10.2174/138945008784533525 18537586

[24] Meeusen EN Snibson KJ Hirst SJ Bischof RJ Sheep as a model species for the study and treatment of human asthma and other respiratory diseases. Drug Discov Today Dis Models. (2009) 6:101–6. 10.1016/j.ddmod.2009.12.002

[25] Plopper CG Mariassy AT Wilson DW Alley JL Nishio SJ Nettesheim P. Comparison of nonciliated tracheal epithelial cells in six mammalian species: ultrastructure and population densities. Exp Lung Res. (1983) 5:281–94. 10.3109/01902148309061521 6662075

[26] Miller HRP. Mucosal mast cells and the allergic response against nematode parasites. Vet Immunol Immunopathol. (1996) 54:331–6. 10.1016/S0165-2427(96)05696-6 8988878

[27] Collie D Pyrah I Watt NJ. Distribution and quantitation of lung parenchymal contractile tissue in ovine lentivirus-induced lymphoid interstitial pneumonia. Do tissue forces limit lung distensibility? Lab Invest. (1995) 73:441–7. 7564278

[28] Matute-Bello G Frevert CW Martin TR. Animal models of acute lung injury. Am J Physiol Lung Cell Mol Physiol. (2008) 295:379–99. 10.1152/ajplung.00010.2008 18621912 PMC2536793

[29] Ackermann MR. Lamb model of respiratory syncytial virus-associated lung disease: insights to pathogenesis and novel treatments. ILAR J. (2014) 55:4–15. 10.1093/ilar/ilu003 24936027 PMC4158344

[30] Viard R Tourneux P Storme L Girard J-M Betrouni N Rousseau J. Magnetic resonance imaging spatial and time study of lung water content in newborn lamb: methods and preliminary results. Invest Radiol. (2008) 43:470–80. 10.1097/RLI.0b013e31816900bb 18496054

[31] Cousens C Scott PR. Assessment of transthoracic ultrasound diagnosis of ovine pulmonary adenocarcinoma in adult sheep. Vet Record. (2015) 177:366–71. 10.1136/vr.103298 26442526

[32] Radu DM Seguin A Bruneval P Fialaire Legendre A Carpentier A Martinod E. Bronchial replacement with arterial allografts. Ann Thorac Surg. (2010) 90:252–8. 10.1016/j.athoracsur.2010.03.079 20609787

[33] Van der Velden J Barker D Barcham G Koumoundouros E Snibson K Assessment of peripheral airway function following chronic allergen challenge in a sheep model of asthma (small airway function in a sheep model of asthma). PLoS ONE. (2011) 6:28740 10.1371/journal.pone.0028740 PMC3236205 22174883

[34] Abraham WM. Modeling of asthma, COPD and cystic fibrosis in sheep. Pulmonary Pharmacol Ther. (2008) 21:743–54. 10.1016/j.pupt.2008.01.010 18339565

[35] Bem RA Domachowske JB Rosenberg HF. Animal models of human respiratory syncytial virus disease. Am J Physiol Lung Cell Mol Physiol. (2011) 301:148–56. 10.1152/ajplung.00065.2011 21571908 PMC3154630

[36] Youssef G Wallace WAH Dagleish MP Cousens C Griffiths DJ. Ovine pulmonary adenocarcinoma: a large animal model for human lung cancer. Insti Lab Anim Res J. (2015) 56:99–115. 10.1093/ilar/ilv014 25991702

[37] DeMartini JC York DF. Retrovirus-associated neoplasms of the respiratory system of sheep and goats: ovine pulmonary carcinoma and enzootic nasal tumor. Vet Clin. (1997) 13:55–70. 9071746 10.1016/s0749-0720(15)30364-9

[38] Palmarini M Fan H Sharp JM. Sheep pulmonary adenomatosis: a unique model of retrovirus associated lung cancer. Trends Microbiol. (1997) 5:478–83. 10.1016/S0966-842X(97)01162-1 9447659

[39] Griffiths DJ Martineau HM Cousens C. Pathology and pathogenesis of ovine pulmonary adenocarcinoma. J Comp Pathol. (2010) 142:260–83. 10.1016/j.jcpa.2009.12.013 20163805

[40] Leroux C Girard N Cottin V Greenland T Mornex J-F Archer F. Jaagsiekte sheep retrovirus (JSRV): from virus to lung cancer in sheep. Vet Res. (2007) 38:211–28. 10.1051/vetres:2006060 17257570

[41] Tustin R Ovine jaagsiekte. J South Afr Vet Med Assoc. (1969) 40:3–23. 5170646

[42] De las Heras M Gonzalez L Sharp J Pathology of ovine pulmonary adenocarcinoma. In: Fan H, editor. Jaagsiekte Sheep Retrovirus and Lung Cancer. Berlin; Heidelberg: Springer (2003). p. 25–54. 10.1007/978-3-642-55638-8_2 12596894

[43] Sanna MP Sanna E De Las Heras M Leoni A Nieddu A Pirino S. Association of jaagsiekte sheep retrovirus with pulmonary carcinoma in Sardinian moufflon (Ovis musimon). J Comp Pathol. (2001) 125:145–52. 10.1053/jcpa.2001.0489 11578130

[44] Caporale M Martineau H De las Heras M Murgia C Huang R Centorame P. Host species barriers to Jaagsiekte sheep retrovirus replication and carcinogenesis. J Virol. (2013) 87:10752–62. 10.1128/JVI.01472-13 23903827 PMC3807380

[45] Fan H. Jaagsiekte Sheep Retrovirus and Lung Cancer. New York, NY: Springer Science & Business Media (2003). 10.1007/978-3-642-55638-810400795

[46] Palmarini M Fan H. Retrovirus-induced ovine pulmonary adenocarcinoma, an animal model for lung cancer. J Natl Cancer Institute. (2001) 93:1603–14. 10.1093/jnci/93.21.1603 11698564

[47] Dungal N Gislason G Taylor E Epizootic adenomatosis in the lungs of sheep-comparisons with jaagsiekte, verminous pneumonia and progressive pneumonia. J Comp Pathol Ther. (1938) 51:46–68. 10.1016/S0368-1742(38)80006-0

[48] Sharp J DeMartini J. Natural history of JSRV in sheep. In: Fan H, editor. Jaagsiekte Sheep Retrovirus and Lung Cancer. Berlin; Heidelberg: Springer (2003). p. 55–79. 10.1007/978-3-642-55638-8_3 12596895

[49] Dungal N Experiments with Jaagsiekte. Am J Pathol. (1946) 22:737–59. 20991974

[50] Grego E De Meneghi D Álvarez V Benito AA Minguijón E Ortín A. Colostrum and milk can transmit jaagsiekte retrovirus to lambs. Vet Microbiol. (2008) 130:247–57. 10.1016/j.vetmic.2008.01.011 18328646

[51] York DF Vigne R Verwoerd DW Querat G. Nucleotide sequence of the jaagsiekte retrovirus, an exogenous and endogenous type D and B retrovirus of sheep and goats. J Virol. (1992) 66:4930–9. 1629959 10.1128/jvi.66.8.4930-4939.1992 PMC241337

[52] Palmarini M Fan H. Molecular biology of jaagsiekte sheep retrovirus. In: Fan H, editor. Jaagsiekte Sheep Retrovirus and Lung Cancer. Berlin; Heidelberg: Springer (2003). p. 81–115. 10.1007/978-3-642-55638-8_4 12596896

[53] Maeda N Palmarini M Murgia C Fan H. Direct transformation of rodent fibroblasts by jaagsiekte sheep retrovirus DNA. Proc Natl Acad Sci USA. (2001) 98:4449–54. 10.1073/pnas.071547598 11296288 PMC31855

[54] Cousens C Maeda N Murgia C Dagleish MP Palmarini M Fan H. *In vivo* tumorigenesis by Jaagsiekte sheep retrovirus (JSRV) requires Y590 in Env TM, but not full-length orfX open reading frame. Virology. (2007) 367:413–21. 10.1016/j.virol.2007.06.004 17610928 PMC2065845

[55] Caporale M Cousens C Centorame P Pinoni C De las Heras M Palmarini M. Expression of the jaagsiekte sheep retrovirus envelope glycoprotein is sufficient to induce lung tumors in sheep. J Virol. (2006) 80:8030–7. 10.1128/JVI.00474-06 16873259 PMC1563803

[56] Wootton SK Halbert CL Miller AD. Sheep retrovirus structural protein induces lung tumours. Nature. (2005) 434:904–7. 10.1038/nature03492 15829964 PMC1401489

[57] Rai SK Duh F-M Vigdorovich V Danilkovitch-Miagkova A Lerman MI Miller AD. Candidate tumor suppressor HYAL2 is a glycosylphosphatidylinositol (GPI)-anchored cell-surface receptor for jaagsiekte sheep retrovirus, the envelope protein of which mediates oncogenic transformation. Proc Natl Acad Sci USA. (2001) 98:4443–8. 10.1073/pnas.071572898 11296287 PMC31854

[58] Zhou S Shoelson SE Chaudhuri M Gish G Pawson T Haser WG SH2 domains recognize specific phosphopeptide sequences. Cell. (1993) 72:767–78. 10.1016/0092-8674(93)90404-E 7680959

[59] Cousens C Alleaume C Bijsmans E Martineau HM Finlayson J Dagleish MP. Jaagsiekte sheep retrovirus infection of lung slice cultures. Retrovirology. (2015) 12:31–47. 10.1186/s12977-015-0157-5 25889156 PMC4419405

[60] De Las Heras M Ortin A Benito A Summers C Ferrer L Sharp J *In-situ* demonstration of mitogen-activated protein kinase Erk 1/2 signalling pathway in contagious respiratory tumours of sheep and goats. J Comp Pathol. (2006) 135:1–10. 10.1016/j.jcpa.2006.02.002 16814801

[61] Maeda N Fu W Ortin A de las Heras M Fan H. Roles of the Ras-MEK-mitogen-activated protein kinase and phosphatidylinositol 3-kinase-Akt-mTOR pathways in Jaagsiekte sheep retrovirus-induced transformation of rodent fibroblast and epithelial cell lines. J Virol. (2005) 79:4440–50. 10.1128/JVI.79.7.4440–44502005 15767444 PMC1061532

[62] Suau F Cottin V Archer F Croze S Chastang J Cordier G. Telomerase activation in a model of lung adenocarcinoma. Eur Respir J. (2006) 27:1175–82. 10.1183/09031936.06.00125105 16455826

[63] Varela M Golder M Archer F de las Heras M Leroux C Palmarini M. A large animal model to evaluate the effects of Hsp90 inhibitors for the treatment of lung adenocarcinoma. Virology. (2008) 371:206–15. 10.1016/j.virol.2007.09.041 17961623 PMC2346560

[64] Arnaud F Caporale M Varela M Biek R Chessa B Alberti A. A paradigm for virus-host coevolution: sequential counter-adaptations between endogenous and exogenous retroviruses. PLoS Pathol. (2007) 3:1716–29. 10.1371/journal.ppat.0030170 17997604 PMC2065879

[65] Cumer T Pompanon F Boyer F. Old origin of a protective endogenous retrovirus (enJSRV) in the Ovis genus. Heredity. (2018) 187–94. 10.1038/s41437-018-0112-z 29976957 PMC6327021

[66] Hecht SJ Carlson JO Demartini JC. Analysis of a type D retroviral capsid gene expressed in ovine pulmonary carcinoma and present in both affected and unaffected sheep genomes. Virology. (1994) 202:480–4. 10.1006/viro.1994.1366 8009860

[67] DeMartini J Carlson J Leroux C Spencer T Palmarini M. Endogenous retroviruses related to jaagsiekte sheep retrovirus. In: Fan H, editor. Jaagsiekte Sheep Retrovirus and Lung Cancer. Berlin; Heidelberg: Springer (2003). p. 117–37. 10.1007/978-3-642-55638-8_5 12596897

[68] Palmarini M Mura M Spencer TE. Endogenous betaretroviruses of sheep: teaching new lessons in retroviral interference and adaptation. J Gen Virol. (2004) 85:1–13. 10.1099/vir.0.19547-0 14718613

[69] Arnaud F Murcia PR Palmarini M. Mechanisms of late restriction induced by an endogenous retrovirus. J Virol. (2007) 81:11441–51. 10.1128/JVI.01214-07 17699582 PMC2045543

[70] Hudachek SF Kraft SL Thamm DH Bielefeldt-Ohmann H DeMartini JC Miller AD. Lung tumor development and spontaneous regression in lambs coinfected with jaagsiekte sheep retrovirus and ovine lentivirus. Vet Pathol Online. (2010) 47:148–62. 10.1177/0300985809352787 20080496

[71] Summers C Norval M De las Heras M Gonzalez L Sharp JM Woods GM. An influx of macrophages is the predominant local immune response in ovine pulmonary adenocarcinoma. Vet Immunol Immunopathol. (2005) 106:285–94. 10.1016/j.vetimm.2005.03.006 15878202

[72] Martineau HM Cousens C Imlach S Dagleish MP Griffiths DJ. Jaagsiekte sheep retrovirus infects multiple cell types in the ovine lung. J Virol. (2011) 85:3341–55. 10.1128/JVI.02481-10 21270155 PMC3067841

[73] Murgia C Caporale M Ceesay O Di Francesco G Ferri N Varasano V Lung adenocarcinoma originates from retrovirus infection of proliferating type 2 pneumocytes during pulmonary post-natal development or tissue repair. PLoS Pathog. (2011) 7:1–12. 10.1371/journal.ppat.1002014 PMC3068994 21483485

[74] Platt J Kraipowich N Villafane F DeMartini J. Alveolar type II cells expressing jaagsiekte sheep retrovirus capsid protein and surfactant proteins are the predominant neoplastic cell type in ovine pulmonary adenocarcinoma. Vet Pathol. (2002) 39:341–52. 10.1354/vp.39-3-341 12014498

[75] De las Heras M de Martino A Borobia M Ortin A Alvarez R Borderias L Solitary tumours associated with Jaagsiekte retrovirus in sheep are heterogeneous and contain cells expressing markers identifying progenitor cells in lung repair. J Comp Pathol. (2014) 15:138–47. 10.1016/j.jcpa.2013.09.001 24176105

[76] Cousens C Bishop JV Philbey AW Gill CA Palmarini M Carlson JO. Analysis of integration sites of Jaagsiekte sheep retrovirus in ovine pulmonary adenocarcinoma. J Virol. (2004) 78:8506–12. 10.1128/JVI.78.16.8506–85122004 15280459 PMC479065

[77] Demartini JC Rosadio RH Lairmore MD. The etiology and pathogenesis of ovine pulmonary carcinoma (sheep pulmonary adenomatosis). Vet Microbiol. (1988) 17:219–36. 10.1016/0378-1135(88)90067-3 3055655

[78] Rosadio R Lairmore M Russell H DeMartini J. Retrovirus-associated ovine pulmonary carcinoma (sheep pulmonary adenomatosis) and lymphoid interstitial pneumonia. I. Lesion development and age susceptibility. Vet Pathol. (1988) 25:475–83. 10.1177/030098588802500611 3212891

[79] Minguijón E González L De las Heras M Gómez N García-Goti M Juste RA. Pathological and aetiological studies in sheep exhibiting extrathoracic metastasis of ovine pulmonary adenocarcinoma (jaagsiekte). J Comp Pathol. (2013) 148:139–47. 10.1016/j.jcpa.2012.06.003 22878053

[80] Nobel T Neumann F Klopfer U. Histological patterns of the metastases in pulmonary adenomatosis of sheep (jaagsiekte). J Comp Pathol. (1969) 79:537–45. 10.1016/0021-9975(69)90074-7 5389142

[81] Martin WB Scott FM Sharp JM Angus KW Norval M. Experimental production of sheep pulmonary adenomatosis (Jaagsiekte). Nature. (1976) 264:183–7. 10.1038/264183a0 186718

[82] Sharp J Angus K Gray E Scott F. Rapid transmission of sheep pulmonary adenomatosis (jaagsiekte) in young lambs. Arch Virol. (1983) 78:89–95. 10.1007/BF01310861 6197047

[83] Salvatori D Gonzalez L Dewar P Cousens C de las Heras M Dalziel RG. Successful induction of ovine pulmonary adenocarcinoma in lambs of different ages and detection of viraemia during the preclinical period. J Gen Virol. (2004) 85:3319–24. 10.1099/vir.0.80333-0 15483246

[84] York DF Vigne R Verwoerd D Querat G. Isolation, identification, and partial cDNA cloning of genomic RNA of jaagsiekte retrovirus, the etiological agent of sheep pulmonary adenomatosis. J Virol. (1991) 65:5061–7. 1651422 10.1128/jvi.65.9.5061-5067.1991 PMC248970

[85] Palmarini M Sharp JM De Las Heras M Fan H. Jaagsiekte sheep retrovirus is necessary and sufficient to induce a contagious lung cancer in sheep. J Virol. (1999) 73:6964–72. 10400795 10.1128/jvi.73.8.6964-6972.1999 PMC112782

[86] DeMartini JC Bishop JV Allen TE Jassim F Sharp JM de las Heras M. Jaagsiekte sheep retrovirus proviral clone JSRVJS7, derived from the JS7 lung tumor cell line, induces ovine pulmonary carcinoma and is integrated into the surfactant protein A gene. J Virol. (2001) 75:4239–46. 10.1128/JVI.75.9.4239–42462001 11287573 PMC114169

[87] Linnerth-Petrik NM Santry LA Darrick LY Wootton SK. Adeno-associated virus vector mediated expression of an oncogenic retroviral envelope protein induces lung adenocarcinomas in immunocompetent mice. PLoS ONE. (2012) 7:51400–15. 10.1371/journal.pone.0051400 23251519 PMC3519541

[88] Palmarini M Sharp JM Lee C Fan H. *In vitro* infection of ovine cell lines by Jaagsiekte sheep retrovirus. J Virol. (1999) 73:10070–8. 10559321 10.1128/jvi.73.12.10070-10078.1999 PMC113058

[89] Archer F Jacquier E Lyon M Chastang J Cottin V Mornex J-F. Alveolar type II cells isolated from pulmonary adenocarcinoma: a model for JSRV expression in vitro. Am J Respir Cell Mol Biol. (2007) 36:534–40. 10.1165/rcmb.2006-0285OC 17158359

[90] Jassim F Sharp J Marinello P. Three-step procedure for isolation of epithelial cells from the lungs of sheep with jaagsiekte. Res Vet Sci. (1987) 43:407–9. 10.1016/S0034-5288(18)30815-4 3444989

[91] Johnson C Fan H. Three-dimensional culture of an ovine pulmonary adenocarcinoma-derived cell line results in re-expression of surfactant proteins and Jaagsiekte sheep retrovirus. Virology. (2011) 414:91–6. 10.1016/j.virol.2011.03.018 21481432 PMC3101266

[92] Sanderson MJ. Exploring lung physiology in health and disease with lung slices. Pulmonary Pharmacol Ther. (2011) 24:452–65. 10.1016/j.pupt.2011.05.001 21600999 PMC3168687

[93] Reynolds SD Hong KU Giangreco A Mango GW Guron C Morimoto Y. Conditional Clara cell ablation reveals a self-renewing progenitor function of pulmonary neuroendocrine cells. Am J Physiol Lung Cell Mol Physiol. (2000) 278:1256–63. 10.1152/ajplung.2000.278.6.L1256 10835332

[94] Kim CFB Jackson EL Woolfenden AE Lawrence S Babar I Vogel S. Identification of bronchioalveolar stem cells in normal lung and lung cancer. Cell. (2005) 121:823–35. 10.1016/j.cell.2005.03.032 15960971

[95] Lundin A Driscoll B. Lung cancer stem cells: progress and prospects. Cancer Lett. (2013) 338:89–93. 10.1016/j.canlet.2012.08.014 22906416 PMC3686996

[96] Archer F Abi-Rizk A Desloire S Dolmazon C Gineys B Guiguen F Lung progenitors from lambs can differentiate into specialized alveolar or bronchiolar epithelial cells. BMC Vet Res. (2013) 9:224–37. 10.1186/1746-6148-9-224 24206786 PMC3831758

[97] Zeltner TB Caduff JH Gehr P Pfenninger J Burri PH The postnatal development and growth of the human lung. Morphometry I. Respir Physiol. (1987) 67:247–67. 10.1016/0034-5687(87)90057-0 3575905

[98] Zeltner TB Burri PH The postnatal development and growth of the human lung. IMorphology I. Respir Physiol. (1987) 67:269–82. 10.1016/0034-5687(87)90058-2 3575906

[99] Nabhan AN Brownfield DG Harbury PB Krasnow MA Desai TJ. Single-cell Wnt signaling niches maintain stemness of alveolar type 2 cells. Science. (2018) 359:1118–23. 10.1126/science.aam6603 29420258 PMC5997265

[100] Zacharias WJ Frank DB Zepp JA Morley MP Alkhaleel FA Kong J. Regeneration of the lung alveolus by an evolutionarily conserved epithelial progenitor. Nature. (2018) 555:251–5. 10.1038/nature25786 29489752 PMC6020060

[101] Scott P Griffiths D Cousens C Diagnosis and control of ovine pulmonary adenocarcinoma (Jaagsiekte). In Practice. (2013) 35:382–97. 10.1136/inp.f4427

[102] Scott P Collie D McGorum B Sargison N. Relationship between thoracic auscultation and lung pathology detected by ultrasonography in sheep. Vet J. (2010) 186:53–7. 10.1016/j.tvjl.2009.07.020 19733102

[103] Cousens C Thonur L Imlach S Crawford J Sales J Griffiths DJ. Jaagsiekte sheep retrovirus is present at high concentration in lung fluid produced by ovine pulmonary adenocarcinoma-affected sheep and can survive for several weeks at ambient temperatures. Res Vet Sci. (2009) 87:154–6. 10.1016/j.rvsc.2008.11.007 19114283

[104] Scott PR Dagleish MP Cousens C. Development of superficial lung lesions monitored on farm by serial ultrasonographic examination in sheep with lesions confirmed as ovine pulmonary adenocarcinoma at necropsy. Irish Vet J. (2018) 71:23–9. 10.1186/s13620-018-0134-0 30450192 PMC6219085

[105] Cousens C Graham M Sales J Dagleish MP. Evaluation of the efficacy of clinical diagnosis of ovine pulmonary adenocarcinoma. Vet Record. (2008) 162:88–95. 10.1136/vr.162.3.88 18204033

[106] Caporale M Centorame P Giovannini A Sacchini F Di Ventura M de las Heras M Infection of lung epithelial cells and induction of pulmonary adenocarcinoma is not the most common outcome of naturally occurring JSRV infection during the commercial lifespan of sheep. Virology. (2005) 338:144–53. 10.1016/j.virol.2005.05.018 15950254

[107] Ortín ME Dewar P García M Ferrer LM Palmarini M Gonzalez L Lack of a specific immune response against a recombinant capsid protein of Jaagsiekte sheep retrovirus in sheep and goats naturally affected by enzootic nasal tumour or sheep pulmonary adenomatosis. Vet Immunol Immunopathol. (1998) 61:229–37. 10.1016/S0165-2427(97)00149-9 9613437

[108] De las Heras M Ortín A Salvatori D de Villareal MP Cousens C Ferrer LM A PCR technique for the detection of Jaagsiekte sheep retrovirus in the blood suitable for the screening of ovine pulmonary adenocarcinoma in field conditions. Res Vet Sci. (2005) 79:259–64. 10.1016/j.rvsc.2005.02.003 16054897

[109] Lewis FI Brülisauer F Cousens C McKendrick IJ Gunn GJ. Diagnostic accuracy of PCR for Jaagsiekte sheep retrovirus using field data from 125 Scottish sheep flocks. Vet J. (2011) 187:104–8. 10.1016/j.tvjl.2009.10.024 19931475

[110] Voigt K Brügmann M Huber K Dewar P Cousens C Hall M. PCR examination of bronchoalveolar lavage samples is a useful tool in pre-clinical diagnosis of ovine pulmonary adenocarcinoma (Jaagsiekte). Res Vet Sci. (2007) 83:419–27. 10.1016/j.rvsc.2007.02.001 17418304

[111] Humann-Ziehank E Renko K Bruegmann ML Devi VR Hewicker-Trautwein M Andreae A. Long-term study of ovine pulmonary adenocarcinogenesis in sheep with marginal vs. sufficient nutritional selenium supply: results from computed tomography, pathology, immunohistochemistry, JSRV-PCR and lung biochemistry. J Trace Elem Med Biol. (2013) 27:391–9. 10.1016/j.jtemb.2013.03.002 23623247

[112] Scott P Cousens C Ultrasonography of ovine pulmonary adenocarcinoma. In Practice. (2018) 40:291–300. 10.1136/inp.k3380

[113] Marland JRK Blair EO Flynn BW González-Fernández E Huang L Kunkler IH Implantable microsystems for personalised anticancer therapy. In: Mitra S Cumming DRS, editors. CMOS Circuits for Biological Sensing and Processing. Cham: Springer International Publishing (2018). p. 259–86. 10.1007/978-3-319-67723-1_11

